# Supplementary material for: MetaRibo-Seq measures translation in microbiomes
Source: Nat Commun. 2020 Jun 29;11:3268. doi: 10.1038/s41467-020-17081-z (PMC7324362; doi:10.1038/s41467-020-17081-z)
Supplement: Supplementary file 10 — Supplementary Data 7 [file 41467_2020_17081_MOESM10_ESM.zip › File2/Confidence_High_Taxonomy/333002_out.krona.html]

Javascript must be enabled to view this page.

members
magnitude
magnitudeUnassigned
count
unassigned
taxon
rank

333002\_out

174

superkingdom
2
174

phylum
1239
173

class
186801
173

173
order
186802

6
family
541000

species
2292180
1

SRS893279\_contig\_number\_12586

species
2292270
1

SRS143181\_contig\_number\_13829

genus
216851
4

4

SRS055982\_contig\_number\_11015SRS063518\_contig\_number\_13467SRS077641\_contig\_number\_1938SRS143148\_contig\_number\_19959
species
853

216572
family
166

genus
459786
166

164

SRS011134\_contig\_number\_22312SRS011302\_contig\_number\_27453SRS013382\_contig\_number\_1048SRS013521\_contig\_number\_12941SRS013687\_contig\_number\_25270SRS013940\_contig\_number\_14452SRS013951\_contig\_number\_20598SRS014235\_contig\_number\_43248SRS014412\_contig\_number\_16021SRS014415\_contig\_number\_contig-100\_868.869SRS014459\_contig\_number\_10186SRS014613\_contig\_number\_179SRS014683\_contig\_number\_4402SRS014855\_contig\_number\_488SRS014923\_contig\_number\_42622SRS014948\_contig\_number\_20449SRS015065\_contig\_number\_contig-100\_1443.206395SRS015579\_contig\_number\_16576SRS015663\_contig\_number\_14735SRS015694\_contig\_number\_17315SRS015782\_contig\_number\_44810SRS015890\_contig\_number\_21648SRS015960\_contig\_number\_20567SRS016018\_contig\_number\_3873SRS016056\_contig\_number\_contig-100\_1609.50962SRS016095\_contig\_number\_23996SRS016335\_contig\_number\_27814SRS016753\_contig\_number\_7788SRS017103\_contig\_number\_35780SRS017191\_contig\_number\_18484SRS017701\_contig\_number\_1499SRS017916\_contig\_number\_11425SRS018351\_contig\_number\_9221SRS018541\_contig\_number\_2224SRS018575\_contig\_number\_7669SRS018623\_contig\_number\_17396SRS018656\_contig\_number\_11248SRS018836\_contig\_number\_815SRS018984\_contig\_number\_27499SRS019030\_contig\_number\_3071SRS019178\_contig\_number\_551SRS019397\_contig\_number\_11223SRS019445\_contig\_number\_1141SRS019496\_contig\_number\_1416SRS019685\_contig\_number\_31011SRS019693\_contig\_number\_986SRS019808\_contig\_number\_11797SRS019910\_contig\_number\_10354SRS019968\_contig\_number\_12624SRS020233\_contig\_number\_49773SRS020271\_contig\_number\_298SRS021484\_contig\_number\_17010SRS021948\_contig\_number\_6229SRS022137\_contig\_number\_16854SRS022609\_contig\_number\_32835SRS024625\_contig\_number\_4572SRS024663\_contig\_number\_9522SRS043411\_contig\_number\_12447SRS043701\_contig\_number\_11404SRS043768\_contig\_number\_27976SRS043841\_contig\_number\_3054SRS044535\_contig\_number\_1509SRS045004\_contig\_number\_30354SRS045713\_contig\_number\_21107SRS045739\_contig\_number\_11131SRS045826\_contig\_number\_12324SRS046502\_contig\_number\_11591SRS047014\_contig\_number\_32327SRS047044\_contig\_number\_4952SRS047433\_contig\_number\_2170SRS047433\_contig\_number\_11686SRS048164\_contig\_number\_30370SRS049446\_contig\_number\_5732SRS049896\_contig\_number\_3817SRS049995\_contig\_number\_15800SRS050422\_contig\_number\_29336SRS050925\_contig\_number\_29111SRS050998\_contig\_number\_13970SRS051031\_contig\_number\_32089SRS053356\_contig\_number\_41136SRS053398\_contig\_number\_13978SRS053573\_contig\_number\_contig-100\_269.37841SRS053649\_contig\_number\_16578SRS054059\_contig\_number\_4638SRS054352\_contig\_number\_3442SRS054905\_contig\_number\_18216SRS055017\_contig\_number\_31310SRS056259\_contig\_number\_22767SRS056273\_contig\_number\_22317SRS056519\_contig\_number\_13436SRS058070\_contig\_number\_15048SRS062701\_contig\_number\_contig-100\_305.56409SRS064276\_contig\_number\_contig-100\_434.192525SRS064757\_contig\_number\_2372SRS065397\_contig\_number\_2659SRS074670\_contig\_number\_5143SRS074964\_contig\_number\_353SRS075078\_contig\_number\_2515SRS075341\_contig\_number\_18918SRS075773\_contig\_number\_25188SRS075821\_contig\_number\_3841SRS075878\_contig\_number\_6949SRS075984\_contig\_number\_16494SRS076756\_contig\_number\_1255SRS076929\_contig\_number\_13009SRS077127\_contig\_number\_15585SRS077231\_contig\_number\_13260SRS077294\_contig\_number\_10826SRS077552\_contig\_number\_20300SRS078176\_contig\_number\_16024SRS078242\_contig\_number\_1307SRS097920\_contig\_number\_7834SRS098073\_contig\_number\_14065SRS098571\_contig\_number\_17432SRS098644\_contig\_number\_911SRS098717\_contig\_number\_3515SRS100907\_contig\_number\_8716SRS103987\_contig\_number\_23397SRS104036\_contig\_number\_6627SRS104084\_contig\_number\_8336SRS1041037\_contig\_number\_13910SRS1041092\_contig\_number\_12412SRS1041134\_contig\_number\_10767SRS1041141\_contig\_number\_14367SRS1041142\_contig\_number\_7197SRS104311\_contig\_number\_29992SRS104327\_contig\_number\_1170SRS104912\_contig\_number\_7542SRS104975\_contig\_number\_2563SRS1055022\_contig\_number\_13498SRS1055083\_contig\_number\_12070SRS1055099\_contig\_number\_7435SRS142542\_contig\_number\_13117SRS142890\_contig\_number\_2187SRS143070\_contig\_number\_16322SRS143085\_contig\_number\_contig-100\_19.52942SRS143342\_contig\_number\_840SRS143417\_contig\_number\_26073SRS143523\_contig\_number\_739SRS143598\_contig\_number\_16761SRS143895\_contig\_number\_33261SRS143991\_contig\_number\_23239SRS144362\_contig\_number\_31804SRS144506\_contig\_number\_25266SRS144603\_contig\_number\_4292SRS145497\_contig\_number\_12922SRS147614\_contig\_number\_2922SRS148253\_contig\_number\_2458SRS148319\_contig\_number\_6266SRS148424\_contig\_number\_17660SRS148721\_contig\_number\_20702SRS148784\_contig\_number\_18180SRS148817\_contig\_number\_795SRS149325\_contig\_number\_14559SRS150029\_contig\_number\_11709SRS893231\_contig\_number\_5070SRS893252\_contig\_number\_8021SRS893292\_contig\_number\_5267SRS893295\_contig\_number\_contig-100\_287.78292SRS893300\_contig\_number\_6515SRS893373\_contig\_number\_14183SRS893378\_contig\_number\_17840SRS893383\_contig\_number\_26482SRS971275\_contig\_number\_22814
species
1945593

species
1897011

SRS062654\_contig\_number\_12461SRS101376\_contig\_number\_11227
2

1950935
species
1

SRS013098\_contig\_number\_24292

1
phylum
976

1
200643
class

1
order
171549

family
171550
1

1
genus
239759

1

SRS147271\_contig\_number\_2286
28117
species
